# Neuroprotective Effects of Some Nutraceuticals against Manganese-Induced Parkinson’s Disease in Rats: Possible Modulatory Effects on TLR4/NLRP3/NF-κB, GSK-3β, Nrf2/HO-1, and Apoptotic Pathways

**DOI:** 10.3390/ph15121554

**Published:** 2022-12-14

**Authors:** Karema Abu-Elfotuh, Ahmed Mohsen Elsaid Hamdan, Asmaa A. Mohammed, Ahmed M. Atwa, Magy R. Kozman, Amany M. Ibrahim, Shaimaa M. Motawea, Heba Mohammed Refat M. Selim, Sally Tohamy Kamal Tohamy, Mahmoud Nour El-Din, Sameh S. Zaghlool, Ayah M. H. Gowifel, Magdy M. Awny

**Affiliations:** 1 Pharmacology and Toxicology Department, Faculty of Pharmacy (Girls), Al-Azhar University, Cairo 11754, Egypt; 2 Pharmacy Practice Department, Faculty of Pharmacy, University of Tabuk, Tabuk 71491, Saudi Arabia; 3 Pharmacology and Toxicology Department, Faculty of Pharmacy, Egyptian Russian University, Cairo 11829, Egypt; 4 Clinical Pharmacy Department, Faculty of Pharmacy, Misr University for Science and Technology, Giza 12563, Egypt; 5 Microbiology and Immunology Department, Sinai University, Arish 45511, Egypt; 6 Howard Taylor Ricketts Laboratory, Department of Microbiology, The University of Chicago, Lemont, IL 60439, USA; 7 Clinical Physiology Department, Faculty of Medicine, Menoufia University, Menoufia 32511, Egypt; 8 Microbiology and Immunology Department, Faculty of Pharmacy (Girls), Al-Azhar University, Cairo 11754, Egypt; 9 Department of Pharmaceutical Sciences, Faculty of Pharmacy, Al-Maarefa University, Ad Diriyah, Riyadh 13713, Saudi Arabia; 10 Pharmacology and Toxicology Department, Faculty of Pharmacy, University of Sadat City (USC), Menoufia 32897, Egypt; 11 Pharmacology and Toxicology Department, Faculty of Pharmacy, Modern University for Technology and Information, Cairo 12055, Egypt; 12 Pharmacology and Toxicology Department, Faculty of Pharmacy, October 6th University, Giza 12585, Egypt

**Keywords:** Parkinson’s disease, manganese, sesamol, thymol, wheat grass, coenzyme Q10, TLR4/NLRP3/NF-κB, GSK3β-Nrf2/HO-1

## Abstract

Parkinson’s disease (PD) is a progressive neurodegenerative disorder affecting the substantia nigra where functions controlling body movement take place. Manganese (Mn) overexposure is linked to a neurologic syndrome resembling PD. Sesamol, thymol, wheat grass (WG), and coenzyme Q10 (CoQ10) are potent antioxidants, anti-inflammatory, and anti-apoptotic nutraceuticals. We investigated the potential protective effects of these nutraceuticals alone or in combinations against MnCl_2_-induced PD in rats. Seven groups of adult male Sprague Dawley rats were categorized as follows: group (I) was the control, while groups 2–7 received MnCl_2_ either alone (Group II) or in conjunction with oral doses of sesamol (Group III), thymol (Group IV), *CoQ10* (Group V), *WG* (Group VI), or their combination (Group VII). All rats were subjected to four behavioral tests (open-field, swimming, Y-maze, and catalepsy tests). Biochemical changes in brain levels of monoamines, *ACHE*, *BDNF*, *GSK-3β*, *GABA*/glutamate, as well as oxidative stress, and apoptotic and neuroinflammatory biomarkers were evaluated, together with histopathological examinations of different brain regions. Mn increased catalepsy scores, while decreasing neuromuscular co-ordination, and locomotor and exploratory activity. It also impaired vigilance, spatial memory, and decision making. Most behavioral impairments induced by *Mn* were improved by sesamol, thymol, *WG*, or *CoQ10*, with prominent effect by sesamol and thymol. Notably, the combination group showed more pronounced improvements, which were confirmed by biochemical, molecular, as well as histopathological findings. Sesamol or thymol showed better protection against neuronal degeneration and some behavioral impairments induced by Mn than WG or CoQ10, partly via interplay between *Nrf2/HO-1, TLR4/NLRP3/NF-κB, GSK-3β* and *Bax/Bcl2* pathways.

## 1. Introduction

Parkinson’s disease (PD) is a chronic neurodegenerative motor disorder that occurs owing to a progressive and intensive dopaminergic (DA) neurons loss in the substantia nigra pars compacta and depletion of dopamine in the striatum, as well as accumulation of alpha-synuclein-containing Lewy bodies throughout the brain [1,2]. In fact, the neuropathogenesis of Parkinson’s disease (PD) is diverse, complicated, and includes several interrelated pathways. Several lines of evidence showed that oxidative stress and neuroinflammation play a critical role in the progression of PD [3,4]. One of the signaling pathways that was notoriously linked to PD neuroinflammation in the brain is the nuclear factor-κB (NF-κB) pathway, which regulates inflammation by controlling the expression of pro-inflammatory genes [5,6]. NF-κB also modulates inflammasome activity leading to the activation of NLRP3 inflammasome and the release of NLRP3-dependent inflammatory cytokines in patients with PD. NLRP3 inflammasome is a multiprotein inflammatory signaling complex, which acts as a molecular driver of the inflammatory response and is activated by a variety of microbial or damage-associated molecular patterns that could emerge from specific cells throughout inflammation, activating Toll-like receptors (TLRs). Of note, the aggregation of α-synuclein protein, which is typical in PD pathogenesis, also activates NLRP3 inflammasome in microglia via interaction with TLRs, where stimulation of TLR can result in amplified NLRP3 transcription and consequent inflammation. The downstream effect of NLRP3 activation includes triggering the activation of caspase-1, which in turn stimulates the maturation and release of interleukin 1β (IL-1β) that mediates the propagation of the inflammatory response [7,8]. Hence, targeting NLRP3 inflammasome-mediated neuroinflammation could be a therapeutic avenue for PD [9]. Additionally, the transcription factor, nuclear factor-erythroid 2-related factor 2 (Nrf2), regulates the transcription of genes encoding protective molecules against oxidative stress, including heme-oxygenase-1 (HO-1), which imparts a neuroprotective effect; thus, the Nrf2/HO-1 signaling pathway plays a pivotal role in guarding against both oxidative and inflammatory damage through inhibition of NF-κB [10]. Another factor that is implicated in the degeneration of dopaminergic neurons characteristic of PD is glycogen synthase kinase-3β (GSK-3β), which plays a critical role in cell apoptosis and neurodegeneration, where GSK-3β activation leads to activation of microglia and an increase in the production of inflammatory cytokines, resulting in neuroinflammation and degeneration. Additionally, abnormal GSK-3β activity may result in phosphorylation of α-synuclein, leading to its aggregation, which is linked with numerous hallmarks of PD. On the other hand, GSK-3β inactivation is indispensable in down-regulating oxidative stress by promoting Nrf2. Thus, hampering GSK-3β activity has become a molecular target for therapeutic amelioration of PD [11]. Taken together, targeting of the TLR4/NLRP3/NF-κB and GSK3β-Nrf2/HO-1 signaling axes may contribute to the inhibition of oxidative stress, neuroinflammation, and apoptosis, hence promoting neuroprotection and exerting potential therapeutic effects against PD. Notably, the typical features of PD, including bradykinesia, resting tremors, rigidity, and postural instability would be developed when 60–70% of dopaminergic neurons in substantia nigra are lost [12,13]. In addition, PD patients often show comorbid non-motor symptoms, such as depression, anxiety, cognitive decline, and olfactory loss, which frequently manifest in the early stages or even during the pre-motor phase of the disease [14]. Furthermore, exposure to environmental neurotoxicants, such as manganese, is strongly linked with PD. Manganese (Mn) is a common environmental pollutant, used in the industrial production of batteries and steel, and also as a fuel additive. It is naturally present in high concentration in some foods, such as nuts and legumes. Thus, humans are regularly exposed to manganese and its salts, leading to high risk of neurodegeneration and precipitation of PD [15]. Interestingly, manganese is a fundamental trace element essential for normal brain and neuronal function, including neurotransmitter synthesis and metabolism [16]. However, exposure to a high dose of Mn may seriously lead to extensive deposition of Mn in specific brain areas, causing neurotoxicity and an extrapyramidal motor disorder known as manganism, a motor dysfunction associated with cognitive and neuropsychiatric deficits similar to parkinsonism [17]. Actually, common mechanisms underlying Mn-induced neurotoxicity include the imbalance between basal ganglia neurotransmitters with consequent neurobehavioral and motor output deficits, which may be related to induction of oxidative stress/inflammation/apoptotic axes in that brain region [18]. Therefore, the halting of hallmark features of PD progression, such as oxidative stress and neuroinflammation may cease this malicious cycle initiated by free radical generation and oxidative status perturbation and ended by apoptosis of basal ganglia neurons.

To date, substantial attention has been paid to search for newer therapeutic approaches against neurodegenerative diseases such as PD, focusing on the use of plant-derived phytochemicals that possess antioxidant and anti-inflammatory properties, with a lesser degree of toxic effects [19,20].

Sesamol (3,4-methylenedioxyphenol) is the major constituent of sesame seed oil (*Sesamum indicum*), possessing beneficial health outcomes, including powerful antioxidant, anti-inflammatory, and anti-mutagenic properties [21,22].

Thymol (2-isopropyl-5-methylphenol) is a dietary monoterpene found predominantly in many edible or culinary plants such as *Nigella sativa*, and Thymus spp. (*Thymus vulgaris*, *Thymus pectinatus*, *Thymus zygis*, and *Thymus ciliates*) [23]. Thymol exhibits potent pharmacological properties, including antioxidant, anti-inflammatory, anti-mutagenic, analgesic, and anti-microbial effects [24,25]

Coenzyme Q10 (CoQ10), a component of the electron transport chain and acting as an antioxidant, is a dietary supplement that is used for preventing neurodegeneration against mitochondrial deficiency and oxidative stress through a reduction in free radical levels [26]. As a potent antioxidant, CoQ10 works by improving the function of mitochondria, which produce energy in cells, and eliminate potentially harmful agents generated during normal metabolism. Moreover, Shults et al. [27] had shown that CoQ10 levels in the mitochondria of PD patients were reduced and that mitochondrial function in these patients was impaired; thus, supplementation of CoQ10 to PD patients significantly increased their blood level of CoQ10.

Wheatgrass (*Triticum aestivum* L.) is a widely used health food, consumed most often as fresh juice or as tablets, capsules, and liquid concentrates. Wheatgrass formulations have been shown to possess various pharmacological properties, such as antioxidant [28] and anti-cancer activities [29].

Although there are remarkable advances in the medical and surgical treatment for PD, definitive disease-modifying therapy is lacking. Hence, developing safe and effective agents from natural medicine for attenuation of PD, as well as the investigation of their molecular mechanisms, have become a research interest. In this regard, the current study aims to demonstrate and compare the possible neuroprotective efficacy and to delineate the underlying mechanistic pathways of sesamol, thymol, CoQ10, or wheat grass either individually or in combination in MnCl_2_-induced rat model of neurodegeneration mimicking PD in humans. In our research, we studied the neuroprotective effect of sesamol, thymol, CoQ10, and Wheatgrass using behavioral, biochemical and histopathological examinations.

## 2. Results

### 2.1. Effect of Sesamol, Thymol, CoQ10, WG, or Their Combination on MnCl_2_-Induced Alterations in Motor Functions in Open-Field Test

**Figure 1:**
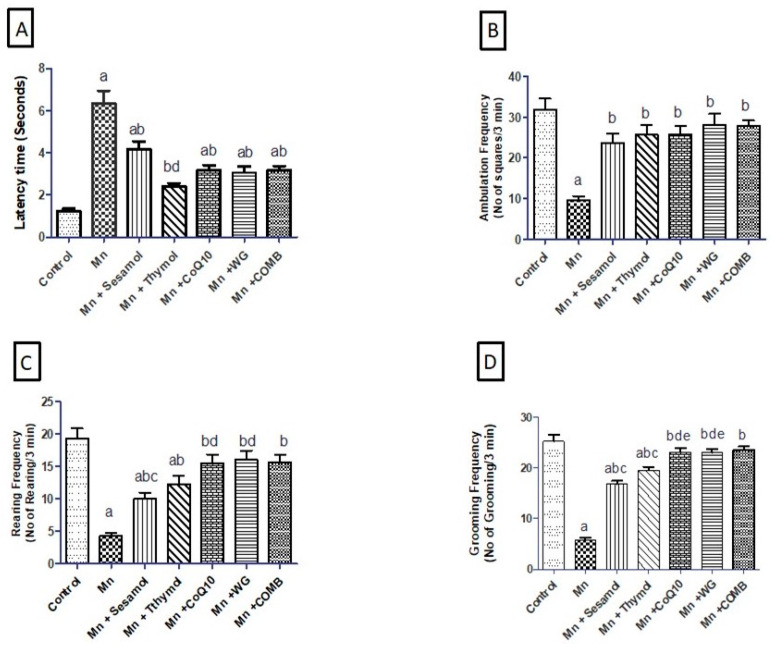
Effects of sesamol, thymol, CoQ10, wheat grass, or their combination on motor functions in open-field test of rats treated with MnCl_2_. (**A**) Latency time, (**B**) Ambulation frequency, (**C**) Rearing frequency, and (**D**) Grooming frequency. Values are means of 12 rats ± S.E.M, as compared with control (a), Mn (b), COMB (c), sesamol (d), and thymol (e) groups. One-way ANOVA with post-test Tukey’s multiple comparison assessed the statistical differences between the various groups, *p*-value < 0.05.

Figure 1 shows that Mn-treated rats exhibited significant decline in animals’ locomotor and exploratory activities in an open-field test. Animals’ ambulation (Figure 1B), rearing (Figure 1C), and grooming frequencies (Figure 1D) decreased markedly following MnCl_2_ administrations by 77.6%, 76.9%, and 69.6%, respectively, compared to the normal control group, while their latency time (Figure 1A) significantly increased by 5-fold as compared to the normal control group. Pretreatment with sesamol, thymol, CoQ10, WG, or their combination exhibited a significant increase in animals’ ambulation frequencies by 145%, 165.6%, 167.4%, 190%, and 188%, rearing by 131%, 185%, 258%, 269% and 261%, and grooming by (188%, 244.4%, 294.5%, 294.5%, and 303%, respectively, along with a significant decrease in their latency time by 34.2%, 61.8%, 50%, 51.3%, and 50%, compared to the MnCl_2_-treated group. It was noticed that CoQ10, WG, or the combination showed a more prominent effect than sesamol in animals’ rearing and produced a more significant effect than sesamol and thymol in animals’ grooming. Meanwhile, thymol elicited the maximum protective effects in the latency test.

### 2.2. Effect of Sesamol, Thymol, CoQ10, WG, or Their Combination on MnCl_2_-Induced Changes in Rats’ Motor, Attention, and Cognitive Functions in Swimming Test and Rats’ Working Memory in the Y-Maze Test

**Figure 2:**
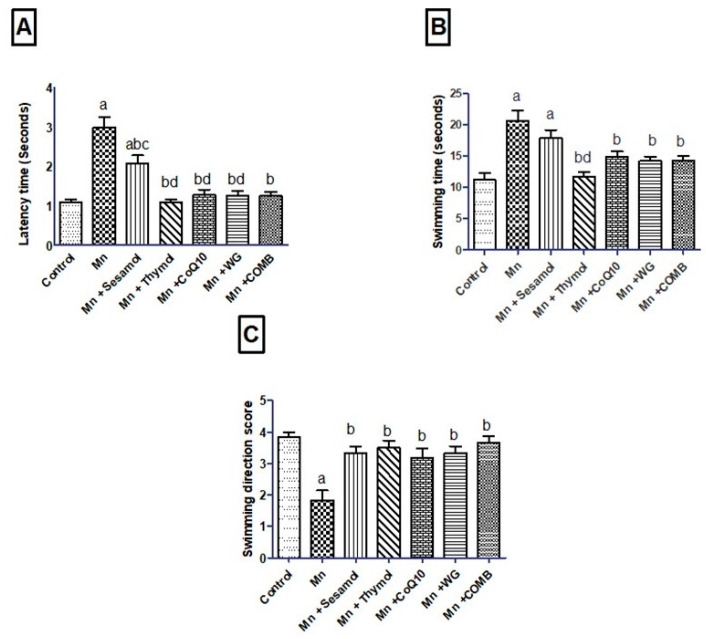
Effects of sesamol, thymol, CoQ10, wheat grass, or their combination on swimming test of rats treated with MnCl_2_. (**A**) latency time, (**B**) swimming time, and (**C**) swimming direction score. Values are means of 12 rats ± S.E.M, as compared with control (a), Mn (b), COMB (c), and sesamol (d) groups. One-way ANOVA with posttest Tukey’s multiple comparison assessed the statistical differences between the various groups, *p*-value < 0.05.

**Figure 3:**
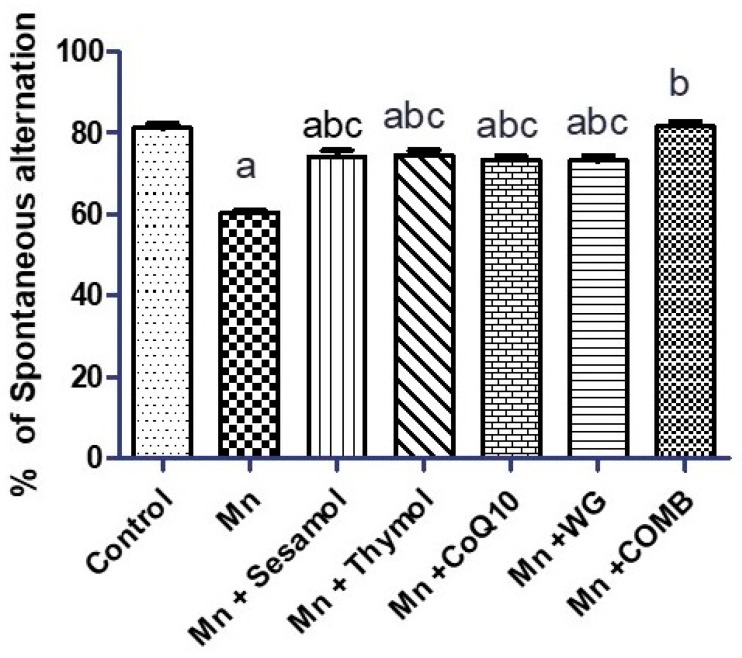
Effects of sesamol, thymol, CoQ10, wheat grass, or their combination on spontaneous alternation percent in Y-maze test of rats treated with MnCl_2_. Values are means of 12 rats ± S.E.M, as compared with control (a), Mn (b), and COMB (c) groups. One-way ANOVA with posttest Tukey’s multiple comparison assessed the statistical differences between the various groups, *p*-value < 0.05.

As depicted in Figure 2, Mn-treated rats exhibited significant decline in animals’ motor activity in the swimming test as evidenced by a significant increase in latency time (Figure 2A) and swimming time (Figure 2B) by 177% and 85%, respectively, compared to the normal control group. However, the swimming score (Figure 2C) declined significantly by 52.2% compared to control rats. Pretreatment with sesamol, thymol, CoQ10, WG, or their combination exhibited a significant decrement in latency time by 30.6%, 64%, 57.3%, 58%, and 58.3% and swimming time by 13.7%, 43.5%, 28.2%, 31.5%, and 30.6%, along with a significant increase in their swimming score by 81.9%, 91.2%, 73%, 81.9%, and 100%, respectively, compared to the MnCl_2_-treated group. Moreover, the combination showed significant reduction in latency time over sesamol monotherapy and succeeded in restoring the working memory to its basal level. As illustrated in Figure 3, rats exposed to MnCl_2_ for 35 days displayed short-term memory deficit, represented by a marked drop in the percentage of spontaneous alternation, recording approximately 0.74-fold as compared to the control rats. Sesamol, thymol, CoQ10, or WG significantly raised the spontaneous alternation by about 1.23, 1.23, 1.21, and 1.21-fold, respectively, as compared with MnCl_2_-treated rats. Interestingly, the combination group completely restored the percentage of spontaneous alternation as compared to monotherapy.

### 2.3. Effect of Sesamol, Thymol, CoQ10, WG, or Their Combination on MnCl_2_-Induced Changes in Catalepsy Scores in Both Bar and Grid Tests

**Figure 4:**
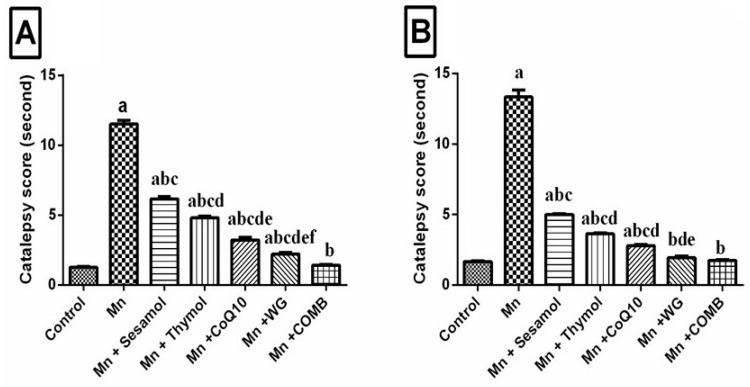
Effects of sesamol, thymol, CoQ10, wheat grass, or their combination on catalepsy score in (**A**) grid and (**B**) bar tests of rats treated with MnCl_2_. Values are means of 12 rats ± S.E.M, as compared with control (a), Mn (b), COMB (c), sesamol (d), thymol (e), and CoQ10 (f) groups. One-way ANOVA with posttest Tukey’s multiple comparison assessed the statistical differences between the various groups, *p*-value < 0.05.

As depicted in Figure 4, Rats exposed to MnCl_2_ showed bradykinesia and rigidity that demonstrated as increased catalepsy score in both the grid test (Figure 4A) and the bar test (Figure 4B) by 9-fold and 8-fold, respectively, as compared with the control group. Pretreatment with sesamol, thymol, CoQ10, or WG significantly reduced the catalepsy duration by 46.5%, 58.3%, 71.9%, and 79.8%, respectively, in the grid test as well as by 62.5%, 72.7%, 79.1%, and 85.3%, respectively, in the bar test, as compared with MnCl_2_-treated rats. In addition, the combination group completely normalized the catalepsy score in both the grid and bar tests. Additionally, the results indicated that the WG treated group revealed marked alteration of catalepsy duration in the grid test and obviously normalized the duration of catalepsy in the bar test.

### 2.4. Effect of Sesamol, Thymol, CoQ10, WG, or Their Combination on MnCl_2_-Induced Changes in Brain Monoamine Neurotransmitter Levels (Dopamine, Norepinephrine, and Serotonin) and ACHE Activity

As illustrated in Table 1, Mn produced significant depletion in brain DA, NE, and 5-HT contents along with a significant elevation in ACHE activity compared to normal rats. Pretreatment with sesamol, thymol, CoQ10, WG, or their combination revealed substantial increments in catecholamine levels by 67.6%, 138.4%, 34.7%, 85%, and 226.3%, respectively, for DA; by 96%, 95.9%, 88.7%, 88.7%, and 138.5%, respectively, for NE; and by 104.5%, 71.6%, 58.9%, 54.5%, and 121.9%, respectively, for 5HT; as well as significant decrease in ACHE activity by 41.1%, 37.6%, 34.1%, 34.1%, and 59.2%, respectively, compared to the MnCl_2_-treated group. The combination group revealed significant improvement in brain DA and NE contents in comparison with monotherapy.

### 2.5. Effect of Sesamol, Thymol, CoQ10, WG, or Their Combination on MnCl_2_-Induced Changes in Cognitive Enhancement Biomarker; BDNF, Neurodegeneration Biomarkers; GABA and Glutamate Levels

As shown in Table 2, administration of MnCl_2_ significantly reduced BDNF and GABA by 62.2% and 76.1%, respectively, but significantly elevated the glutamate level by 663.6% as compared to the normal control group. However, pretreatment with sesamol, thymol, CoQ10, WG, or their combination significantly increased BDNF by 98.6%, 100.8%, 100.6%, 70.3%, and 127.6%; GABA by 62.52%, 93.7%, 55.4%, 55.4% and 166.9%, respectively, but significantly decreased the glutamate level by 51.1%, 42.8%, 38.1%, 38.1% and 53.6%, respectively, as compared to the MnCl_2_-treated group. Interestingly, the combination of these tested agents demonstrated a significant improvement in the levels of BDNF and GABA as compared to monotherapy.

### 2.6. Effect of Sesamol, Thymol, CoQ10, WG, or Their Combination on MnCl_2_-Induced Changes in Brain Redox Status and Oxidative Stress Biomarkers; Nrf2, HO-1, and TAC

**Figure 5:**
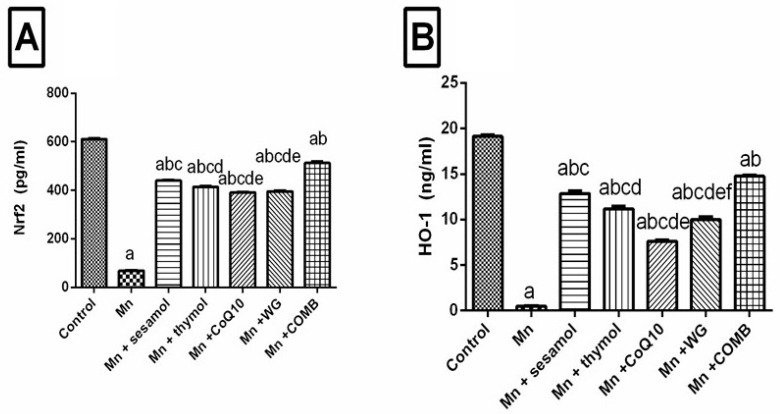
Effects of sesamol, thymol, CoQ10, wheat grass, or their combination on MnCl_2_-induced changes in (**A**) Nuclear Factor Erythroid 2-Related Factor 2 (Nrf2) and (**B**) Hemoxygenase-1 (HO-1). Values are means of 6 rats ± S.E.M, as compared with control (a), Mn (b), COMB (c), sesamol (d), thymol (e), and CoQ10 (f) groups. One-way ANOVA with posttest Tukey’s multiple comparison assessed the statistical differences between the various groups, *p*-value < 0.05.

As illustrated in Figure 5 and Table 3, rats exposed to manganese showed significant decreases in their brain Nrf2 (Figure 5A) and HO-1 (Figure 5B) by 88.7% and 97.4%, respectively, and TAC (Table 3) contents as well as SOD (Table 3) activity by 80.9% and 94.4%, respectively, as compared to the control group. On the other hand, MDA level and iNOS (Table 3) activity were significantly increased in MnCl_2_-exposed rats by 1222% and 2637%, respectively, as compared to the control group. Pretreatment of rats with sesamol, thymol, CoQ10, WG, or their combination demonstrated considerable increments in levels of redox markers by 539.3%, 501.5%, 467.2%, 474.3%, and 644.7%, respectively, for Nrf2; by 2468%, 2134%, 1416%, 1904%, and 2854%, respectively, for HO-1; by 202.2%, 143.8%, 183.7%, 104.5%, and 273%, respectively, for TAC; and by 775%, 725%, 600%, 625%, and 1125, respectively for SOD, as well as significant decrements in both MDA level by 44.5%, 51.2%, 29.3%, 30.7%, and 58%, respectively; and iNOS activity by 67.8%, 66.6%, 58.2%, 51.6%, and 83.6%, respectively, compared to the MnCl_2_-control group. The combination group revealed significant improvement in brain Nrf2, HO-1, TAC contents, and SOD activity, as well as significant reduction in iNOS activity compared to monotherapy.

### 2.7. Effects of Sesamol, Thymol, CoQ10, WG, or Their Combination on MnCl_2_-Induced Changes in Brain Inflammatory Biomarkers; TLR4, NLRP3, NF-κB, Caspase-1 and Cox-2 and Pro-Inflammatory Cytokines; TNF-α, IL-1β

**Figure 6:**
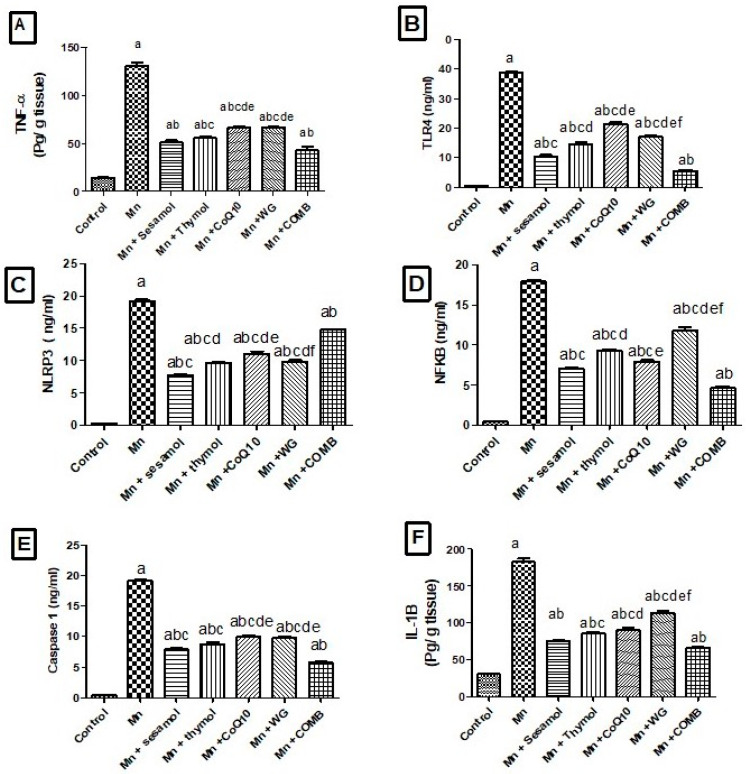
Effects of sesamol, thymol, CoQ10, wheat grass, or their combination on brain inflammatory markers in rats treated with MnCl_2_. (**A**) tumor necrosis factor alpha, (**B**) TLR4, (**C**) NLRP3, (**D**) NF-κB, (**E**) Caspase-1, and (**F**) IL-1β. Values are means of 8 rats ± S.E.M, as compared with control (a), Mn (b), COMB (c), sesamol (d), thymol (e), and CoQ10 (f) groups. One-way ANOVA with posttest Tukey’s multiple comparison assessed the statistical differences between the various groups, *p*-value < 0.05.

As depicted in Figure 6 and Table 2, MnCl_2_ triggered inflammation via significantly elevating the brain TNF-α (Figure 6A), TLR4 (Figure 6B), NLRP3 (Figure 6C), NF-κB (Figure 6D), caspase-1 (Figure 6E), and IL-1β (Figure 6F) contents by 9.1, 97.2, 60.1, 44.75, 51.6, and 6-fold, respectively, together with the COX-2 (Table 2) level in comparison with the control rats. However, sesamol, thymol, CoQ10, WG, or their combination significantly reversed such increments in TNF-α level by 61%, 57.4%, 49%, 49%, and 67.3%, respectively; TLR4 level by 73.3%, 62.2%, 44.8%, 56.3%, and 85.8%, respectively; NF-κB level by 60.8%, 48.5%, 55.6%, 34.4%, and 74.3%, respectively; NLRP3 level by 59.9%, 49.7%, 42.7%, 48.9%, and 74.2%, respectively; caspase-1 level by 58.5%, 54.1%, 47.6%, 48.8%, and 69.6%, respectively; IL-1β level by 58.5%, 53%, 50.5%, 37.7%, and 63.4%, respectively; and COX-2 contents by 56.1%, 53.6%, 53.4%, 52.7%, and 36.1%, respectively, compared with the MnCl_2_-control group, with the highest effect for the sesamol-treated group. Of note, the results indicated that the effect of combination group on TNF-α is more pronounced than thymol, WG, or CoQ10 monotherapy. In addition, combination group significantly reduced TLR4, NF-Kβ, caspase-1, and IL-1β levels as compared with monotherapy.

### 2.8. Effects of Sesamol, Thymol, CoQ10, WG, or Their Combination on MnCl_2_-Induced Changes in Brain Apoptotic Biomarkers; Bax and Bcl2

**Figure 7:**
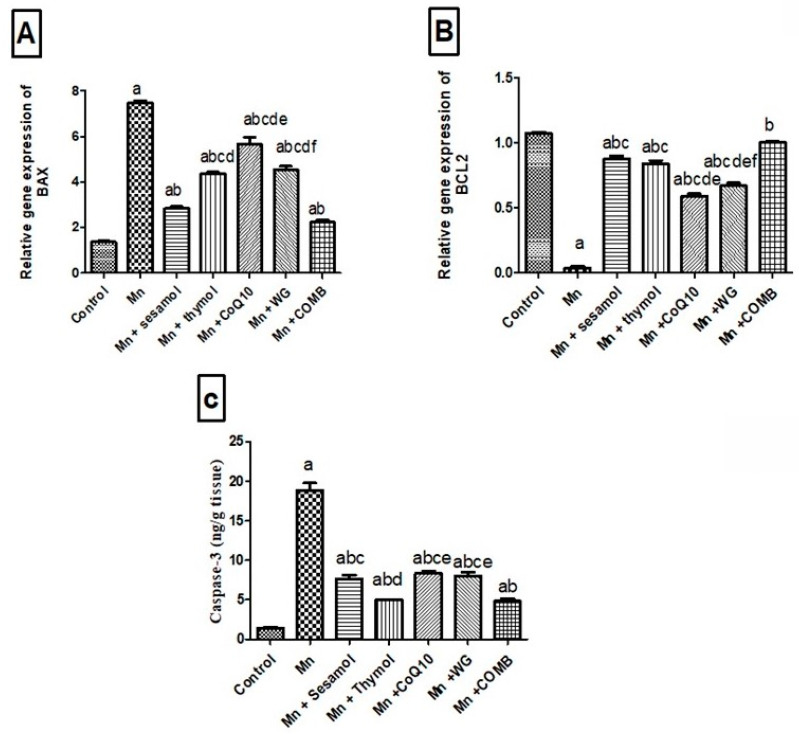
Effects of sesamol, thymol, CoQ10, wheat grass, or their combination on MnCl_2_-induced changes in (**A**) *Bax* mRNA expression level, (**B**) *Bcl2* mRNA expression level, and (**C**) caspase-3 protein content. Values are means of 8 rats ± S.E.M, as compared with control (a), Mn (b), COMB (c), sesamol (d), thymol (e), and CoQ10 (f) groups. One-way ANOVA with posttest Tukey’s multiple comparison assessed the statistical differences between the various groups, *p*-value < 0.05.

As illustrated in Figure 7, the MnCl_2_-exposed group showed a significant elevation in the brain mRNA expression level of the proapoptotic marker, *Bax* (Figure 7A), and a significant decrease in brain mRNA level of the anti-apoptotic marker, *Bcl2* (Figure 7B), by 443.5% and 96.6%, respectively, compared to the normal control group. Additionally, MnCl_2_ significantly elevated *caspase-3* (Figure 7C) level by 1218% compared to the normal control group. Pretreatment with sesamol, thymol, WG, or CoQ10 markedly increased *Bcl2* mRNA expression level by 22, 21, 15, and 17-fold, respectively, compared to the MnCl_2_-control group with highest effect for sesamol. In addition, the combination group brought *Bcl2* mRNA expression level back to normal compared to MnCl_2_-control rats. On the other hand, mRNA expression level of *Bax* significantly decreased in all treated groups (sesamol, thymol, CoQ10, WG, or their combination) by 62%, 40.5%, 23%, 61.5%, and 68.9%, respectively compared to the MnCl_2_-control rats. Similarly, caspase-3 level markedly decreased in all treated groups (sesamol, thymol, CoQ10, WG, or their combination) by 59.6%, 73.6%, 55.6%, 57.4%, and 74.4%, respectively, compared to MnCl_2_-control rats.

### 2.9. Effect of Sesamol, Thymol, CoQ10, WG, or Their Combination on MnCl_2_-Induced Changes in Astroglial Injury Biomarker; GFAP, Tissue Injury Biomarker; AIF and Cognitive Impermanent Biomarker; GSK-3β mRNA Expression

**Figure 8:**
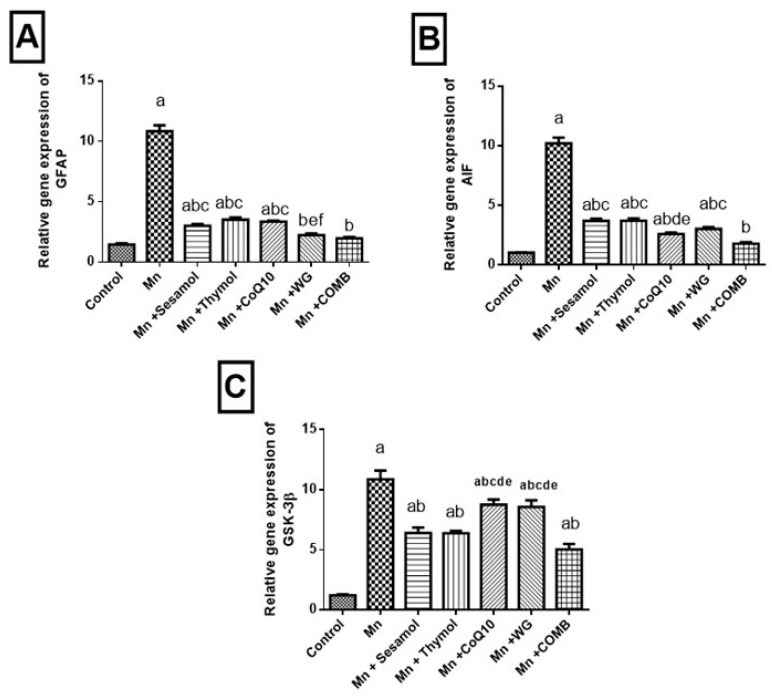
Effects of sesamol, thymol, CoQ10, wheat grass, or their combination on MnCl_2_-induced changes in mRNA expression levels of (**A**) Glial fibrillary acidic protein (GFAP), (**B**) apoptosis inducing factor (AIF), and (**C**) glycogen synthase kinase 3-beta (GSK-3β). Values are means of 6 rats ± S.E.M, as compared with control (a), Mn (b), COMB (c), sesamol (d), thymol (e), and CoQ10 (f) groups. One-way ANOVA with posttest Tukey’s multiple comparison assessed the statistical differences between the various groups, *p*-value < 0.05.

As illustrated in Figure 8, the MnCl_2_ exposure caused an elevation in the brain mRNA expression of *GSK-3β*, *GFAP*, and *AIF* that were raised to 8.9, 7.45, and 9.7-fold, respectively, relative to the normal control rats. On the contrary, pretreatment with sesamol, thymol, or CoQ10 hampered the mRNA expression of *GFAP* by 72.17%, 67.53%, and 69%, respectively, compared to MnCl_2_-control rats. In addition, WG and combination groups brought *GFAP* mRNA expression level back to normal compared to MnCl_2_-control rats. Additionally, *AIF* mRNA expression was reduced in sesamol, thymol, CoQ10, or WG groups by 63.65%, 63.56%, 74.46%, and 70.32%, respectively, compared to MnCl_2_-control rats. Furthermore, in the combination group *AIF* expression was normalized as compared to MnCl_2_-control rats. However, pretreatment with sesamol, thymol, CoQ10, WG, or their combination halted *GSK-3β* expression by 41.15%, 41.33%, 19.58%, 21.14%, and 53.69%, respectively, compared to MnCl_2_-control rats.

### 2.10. Effect of Sesamol, Thymol, CoQ10, WG, or Their Combination on MnCl_2_-Induced Histopathological Alterations in Brain Tissues

**Figure 9:**
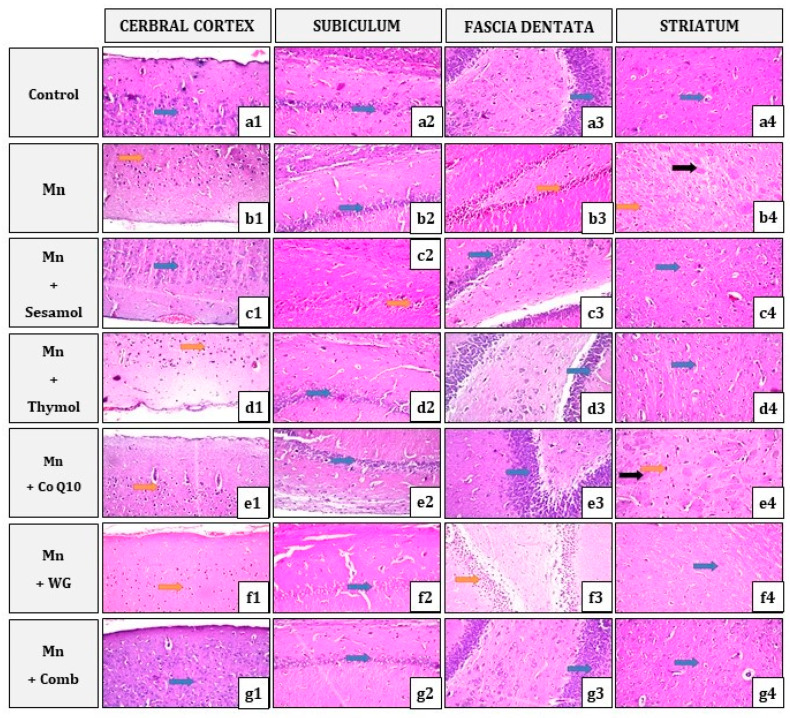
Photomicrographs of brain sections (cerebral cortex, subiculum and fascia dentata in hippocampus, and striatum areas) stained by Hematoxylin and Eosin (magnification power of 40×). Where: (**a1**–**a4**): control group, (**b1**–**b4**): Mn group, (**c1**–**c4**): Mn + Sesamol group, (**d1**–**d4**): Mn + Thymol group, (**e1**–**e4**): Mn + CoQ10 group, (**f1**–**f4**): Mn + WG group, and (**g1**–**g4**): Mn + Combination of Sesamol + Thymol + CoQ10 + WG. Where: (**blue arrow**) indicates no histopathological alteration, (**orange arrow**) indicates nuclear pyknosis and degeneration, and (**black arrow**) indicates focal eosinophilic plagues.

As depicted in Figure 9, brain tissues including the cerebral cortex, subiculum and fascia dentata in hippocampus, and striatum areas, of the normal control group showed normal architecture without abnormal histological alterations. On the other hand, in the Mn-treated group, the neuronal cells of the cerebral cortex, as well as the fascia dentate and hilus of the hippocampus, showed nuclear pyknosis and degeneration. Moreover, multiple focal eosinophilic plagues were found in the striatum. All the aforementioned histological alterations were greatly ameliorated by variable degrees upon treatment with sesamol, thymol, CoQ10, WG, or their combination. The group treated with the combination achieved the best results with apparent normal histological picture of the brain tissues.

In the control group, there was no histopathological alteration in the neuronal cells of the cerebral cortex, hippocampus (subiculum, fascia dentate, and hilus) and striatum areas (Figure 9(a1–a4). In the Mn group, Nuclear pyknosis and degeneration were detected in the neurons of the cerebral cortex (Figure 9(b1)). There was no alteration in the neurons of the subiculum of the hippocampus (Figure 9(b2)). Nuclear pyknosis was also noticed in some neurons of the fascia dentate and hilus of the hippocampus (Figure 9(b3)). The striatum showed the formation of multiple focal eosinophilic plagues, with nuclear pyknosis and degeneration in some neurons (Figure 9(b4)). In the Mn + Sesamol group, the neurons in the cerebral cortex showed intact histological structure (Figure 9(c1)) while the other neurons in the subiculum in the hippocampus had degeneration and nuclear pyknosis (Figure 9(c2)). There was no alteration in the neurons of the fascia dentate and hilus of the hippocampus (Figure 9(c3)) as well as the striatum (Figure 9(c4)). In the Mn + Thymol group, Nuclear pyknosis and degeneration were detected in the neurons of the cerebral cortex (Figure 9(d1)). There was no alteration in the neurons of the subiculum, fascia dentate of the hippocampus (Figure 9(d2,d3)), and striatum (Figure 9(d4)). In the Mn + Co Q10 group, the neurons of the cerebral cortex showed nuclear pyknosis and degeneration (Figure 9(e1)). There was no histopathological alteration in the neurons of the hippocampus (subiculum and fascia dentate) (Figure 9(e2,e3)). The striatum showed the formation of multiple focal eosinophilic plagues, with nuclear pyknosis and degeneration in some neurons (Figure 9(e4)). In the Mn + WG group, the cerebral cortex showed nuclear pyknosis and degeneration, which were detected in all the neurons (Figure 9(f1)). There was no histopathological alteration recorded in the subiculum area of the hippocampus (Figure 9(f2)). There was nuclear pyknosis and degeneration observed in all the neurons of the fascia dentata of the hippocampus (Figure 9(f3)). There was no histopathological alteration recorded in the striatum area (Figure 9(f4)). In the Mn + Combination of Sesamol + Thymol + CoQ10 + WG group, there was no histopathological alteration in the cerebral cortex (Figure 9(g1)), subiculum (Figure 9(g2)), fascia dentate and hilus of the hippocampus (Figure 9(g3)), and the striatum (Figure 9(g4)).

## 3. Discussion

This study investigated the effect of prolonged exposure to MnCl_2_ on oxidative stress and neuroinflammatory and apoptotic pathways, together with the motor and behavioral activities in rats. There is ample evidence reporting the therapeutic benefits of phytochemicals in treating many neurodegenerative diseases, including PD [30,31,32,33,34,35,36,37]. Hence, the aim of the current study was to investigate the possible neuroprotective effects of sesamol, thymol, CoQ10, or WG, either individually or in combination with each other on MnCl_2_-induced behavioral, molecular, and neurochemical alterations that may be correlated with motor dysfunction of parkinsonism-like conditions. To the best of our knowledge, this is the first study that compares the neuroprotective potentials of these natural agents to slacken the progression of PD in rats.

Previous studies showed that prolonged exposure to MnCl_2_ resulted in degeneration of dopaminergic neurons, which is followed by behavioral, neurochemical, and neuropathological alterations that closely mimic those of PD in humans, where motor dysfunction in humans and rodents was observed following MnCl_2_ administration [18,32,33]. These observations lend support to ours, where the current results showed that induction of PD by MnCl_2_ administration was associated with an increase in the catalepsy scores of grid and bar tests, as well as an increase in the latency of open-field and swimming tests, compared to normal control rats, suggesting the elaboration of bradykinesia, rigidity, and motor deficits in rats under investigation. In addition, the current study showed that exposure to MnCl_2_ resulted in impairment in spatial working memory as evidenced by a decline in cognitive functions in the Y-maze test compared to normal control rats. Contrariwise, the administration of sesamol, thymol, CoQ10, WG, or their combination attenuated the MnCl_2_-induced catalepsy and restored the impaired locomotor performance and cognitive functions. These observations were verified by earlier studies [34,35,36]. The combination group showed better outcomes in comparison to monotherapy. Moreover, the present study demonstrated that MnCl_2_ exposure mediated an impairment in learning, memory, and locomotion, which may be attributed to its effect on the cholinergic system [37], as evidenced by the depicted significant elevation in ACHE activity compared to the normal control group. These alterations were averted by all administered drugs with the maximal effect in the combination group. It has been reported that the interrelated pathways of oxidative perturbations, mitochondrial dysfunction, protein aggregation, neuroinflammation, and apoptotic death of dopaminergic neurons may contribute to the pathophysiology of PD [38,39].

In the current work, MnCl_2_-exposed rats exhibited a marked degeneration of dopaminergic neurons in the nigrostriatal area that was evidenced by a significant decrease in the brain monoamines (DA, NE, and 5-HT) levels compared to the normal control group, results that comes in line with those previously reported by Kwakye et al. [33]. In addition, MnCl_2_-treated rats showed a significant decrease in the level of BDNF compared to the normal control group, where BDNF is the key molecule in regulating not only neuronal development, differentiation, maintenance, and survival, but also in regulating cognitive function. These results are in agreement with previous studies [40,41], confirming the crucial role of the DA/BDNF axis in PD etiopathogenesis. Additionally, rats exposed to MnCl_2_ demonstrated marked GABA/Glutamate imbalance compared to normal rats, resulting in excitotoxicity that occurs as a consequence of dopamine depletion and glutamate-mediated increase in levels of free cytosolic calcium, which causes mitochondrial damage and formation of oxidizing species [42]. On the other hand, pretreatment with sesamol, thymol, CoQ10, WG, or their combination significantly elevated brain levels of monoamines (DA, NE and 5-HT) and BDNF, reflecting a neurotrophic property, compared to MnCl_2_-control rats, with the highest reading demonstrated by the combination group. These results suggested the complementary neuroprotective effect of these antioxidants, which was evidenced by the restoration of dopaminergic transmission, together with the reduction in ACHE activity and hence improvement in the motor performance and cognitive function of MnCl_2_-exposed rats.

Oxidative stress has been considered as a cornerstone in Mn-induced neurotoxicity, where excessive reactive oxygen species (ROS) production, associated with depleted antioxidant defense mechanisms, may account for the increased oxidative damage to the brain, which is particularly vulnerable to oxidative damage, owing to its high content of polyunsaturated fatty acids [18,43]. The results of the current work demonstrated that long-term exposure to MnCl_2_ resulted in perturbation of brain redox status, where Nrf2, HO-1, and TAC levels as well as SOD activity were hampered, while MDA and iNOS levels were elevated, compared to the normal control group. These results reflected a state of oxidative stress that is associated with aberrant activation of *GSK-3β*, which inhibits gene regulatory function of *Nrf2*, and this in turn downregulates the expression of antioxidant genes, such as *HO-1* [44]; results that are quite related to those of the current study. In this context, augmenting the intrinsic antioxidant defense is one of the successful strategies that can achieve neuroprotection against PD animal models [45]. In the current study, pretreatment with sesamol, thymol, CoQ10, WG, or their combination significantly enhanced the brain antioxidant defenses, evidenced by the improved levels of TAC, Nrf2, HO-1, and SOD activity, as well as the lessened activity of iNOS and MDA content. These results suggested that the aforementioned treatments mitigated MnCl_2_-induced oxidative damage in the brain tissues by virtue of their potent antioxidant and/or free radical scavenging properties [46,47]. Additionally, these findings may be related to inhibition of *GSK-3β* and the activation of the Nrf2/HO-1 pathway [48].

Of note, neuroinflammation has been recognized as a major culprit in the pathogenesis of PD in various experimental models of PD, including manganese-induced PD [18,49]. Actually, the generation of ROS induces inflammation through the activation of IκB kinase (IKK), with consequent NF-κB activation, leading to overproduction and release of its downstream pro-inflammatory mediators from activated glial cells in the brain [50,51]. In addition, it has been reported that α-syn protein aggregates can activate NLRP3 inflammasome in microglia through an interaction with TLRs and activation of NF-kB [52,53]. Upon activation, NLRP3 associates with caspase-1 and the adaptor molecule apoptosis-associated speck-like protein containing a caspase recruitment domain (ASC) to foster caspase-1 activation, that, in turn, cleaves pro-IL-1β into its biologically active form [54,55]. This possibly contributes to neuroinflammation and neuronal death [56]. These explanations strengthened our results, which showed that MnCl_2_-exposed rats exhibited heightened inflammatory response, as evidenced by the significant spike in brain levels of TLR4, NLRP3 inflammasome, caspase-1, and NF-kB, along with the levels of its downstream pro-inflammatory mediators including TNF-α, IL-1β, COX2, and iNOS, compared to normal control rats. This ongoing neuroinflammation may be linked to the depicted upregulation in *GSK-3β* expression level in MnCl_2_-exposed rats, as reported in the current study. Of note, the activation of *GSK-3β* can promote neuroinflammation by activating microglia, together with enhancing the production of inflammatory cytokines, which can in turn exacerbate neuronal damage with progressive loss of nigral dopaminergic neurons [20,50]. Remarkably, MnCl_2_-exposed rats showed an increased mRNA expression of *GFAP*, compared to normal control rats, where GFAP is a protein released by activated astroglia, which plays a key role in neuroinflammation and neurodegeneration in PD [57]. Conversely, rats treated with sesamol, thymol, CoQ10, WG, or their combination exhibited marked anti-inflammatory effects evidenced by reduced brain levels of TLR4, NLRP3 inflammasome, caspase-1, and NF-kB, and TNF-α, IL-1β, COX2 and iNOS, together with the mRNA expression of *GFAP* compared to MnCl_2_-control rats. These effects may be attributed to the depicted inhibition of GSK-3β mRNA expression by all these aforementioned treatments by variable degrees, as documented in the current study, where the maximal effect was noticed in the combination group in the majority of these measured parameters. These observations are in line with those of [47,58].

Notably, the major contributors in Mn-induced neurotoxicity are not limited to the crosstalk between neuroinflammation and oxidative stress, but extended to include neuronal apoptosis of dopaminergic neurons, as one of the major neurotoxic mechanisms induced by MnCl_2_ [18,41]. In the current work, Mn-exposed rats depicted a remarkable elevation in *Bax* mRNA expression, *AIF* mRNA expression, and caspase-3 content, along with marked reduction in *Bcl2* mRNA expression, compared to the normal control group. These findings may be linked to the depicted elevation in the mRNA expression of *GSK-3β* by MnCl_2_. It had been reported that the activation of GSK-3β modulates several apoptotic signals by increasing *Bax* expression, together with its translocation from the cytosol to the mitochondria, and promoting the release of cytochrome c, thus causing apoptosis of brain dopaminergic neurons [59]. On the contrary, rats treated with sesamol, thymol, CoQ10, WG, or their combination exhibited upregulation of *Bcl2* mRNA expression, coupled with significant reduction in *Bax* mRNA expression, *AIF* mRNA expression, and caspase-3 content compared to MnCl_2_-control rats. These effects may be related to inhibition of *GSK-3β* activity by the tested agents, where inhibiting *GSK-3β* is suggested to have a protective effect on dopaminergic neurons against dopaminergic neurodegeneration by hampering the oxidative stress and promoting the increased expression of *Bcl2*, which abrogates the neuronal cell apoptosis associated with PD [59]. These are results that are corroborated by previous studies [60,61,62,63,64,65,66,67,68].

It is also important to mention that all these previous biochemical findings were further confirmed by histopathological findings. Histopathological examination of H&E-stained sections of animals’ cortexes, hippocampi, and striatums following MnCl_2_ exposure depicted several histopathological changes, including nuclear pyknosis and degeneration in the neurons of the cerebral cortex and the fascia dentate and hilus of the hippocampus. In addition, the striatum of MnCl_2_-control rats showed formation of multiple focal eosinophilic plagues with nuclear pyknosis and degeneration in some neurons. On the other hand, pretreatment with sesamol showed improvement in these alterations in the different areas of the brain, except some neurons in the subiculum in the hippocampus had degeneration and nuclear pyknosis. Moreover, thymol-treated rats showed no histopathological alterations in the neurons of the hippocampus (subiculum and fascia dentate) and striatum, but showed nuclear pyknosis and degeneration in the neurons of the cerebral cortex. Additionally, the CoQ10-treated group showed no histopathological alteration in the neurons of the hippocampus (subiculum and fascia dentate), whereas it showed nuclear pyknosis and degeneration in some neurons of the cerebral cortex and the striatum. Additionally, WG-treated animals revealed no histopathological alteration only in the subiculum area of the hippocampus and the striatum, but the cerebral cortex and the fascia dentata of the hippocampus showed nuclear pyknosis and degeneration. However, the combination group achieved the best results, where the cerebral cortex, the hippocampus, and the striatum depicted no histopathological alterations.

## 4. Materials and Methods

### 4.1. Animals

Eighty-four adult male Sprague Dawley rats (weighting 300–320 g, and aged 8 months old) were purchased from the Nile Co. for Pharmaceuticals and Chemical Industries, Cairo, Egypt and acclimatized for one week before the experiment started. The animals were supplied with standard diet pellets (El-Nasr Chemical Co., Abu Zaabal, Cairo, Egypt) and water was given ad libitum. The rats were housed in stainless-steel cages (three rats/cage) and kept at the animal house facility, Faculty of Pharmacy, Al-Azhar University “girls”, under standard housing conditions (temperature of 25 ± 1 °C and humidity (50 ± 5%)) with 12 h light and dark cycles. Animal experiments were usually carried out at a fixed time around 8 a.m.–2 p.m. All animal procedures and the experimental protocols were approved by the Animal Ethics Committee of the Faculty of Pharmacy, Al-Azhar University, Egypt (Ethical approval No. 215/2021) and complied with the Guide for the Care and Use Laboratory Animals published by the National Institutes of Health (NIH Publications No. 8023, revised 1978).

### 4.2. Drugs and Chemicals

Manganese (Mn) as manganese (II) chloride tetrahydrate (MnCl_2_.4H_2_O), was purchased from Sigma-Aldrich (St. Louis, MO, USA) and was freshly dissolved in normal saline (NaCl; 0.9%, “El-Nasr”) [68]. Sesamol, thymol, and CoQ10 were purchased from Sigma-Aldrich (St. Louis, MO, USA) and were suspended in 1% tween 80 in normal saline [63,64,65]. Wheatgrass (WG) was purchased from Bioglan superfoods, UK and was freshly prepared, suspended in 1% tween in normal saline. The chemical constituents of WG were previously identified and analyzed using high-performance liquid chromatography (HPLC) in our previous work as described by [66]. All other chemicals and solvents used in the current study were of the highest grade commercially available.

### 4.3. Experimental Design

**Figure 10:**
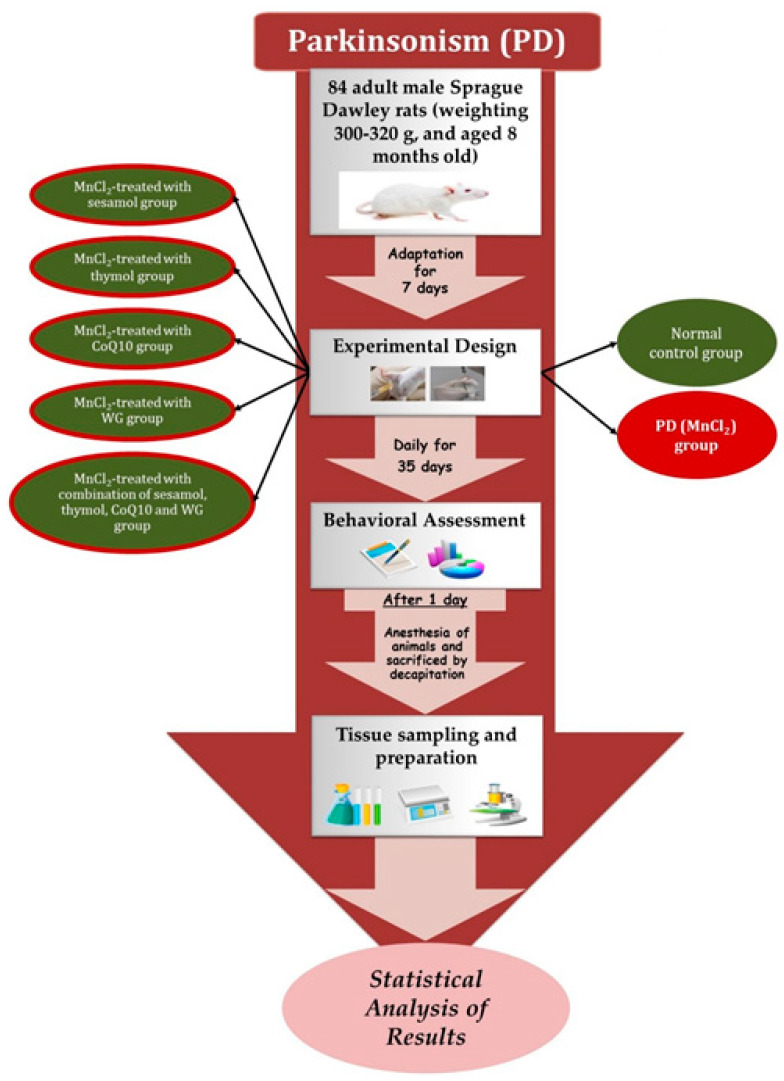
Summary of the experimental design and all behavioural and biochemical tests.

Eighty-four adult male Sprague Dawley rats were randomly allocated into seven groups (*n* = 12) as follows (Table 4):

The rats were allowed to acclimatize for 1 week before starting the study which spanned over a total period of 35 days. At the end of the study, after five weeks (Day 35), 24 h after the last MnCl_2_ dose for each group, animals’ behavioral tests were conducted. After 24 h from behavioral tests, rats were anesthetized by injection of ketamine (80 mg/kg, i.p.) and euthanized for tissue sampling to perform biochemical analyses and histopathological examination (Figure 10).

### 4.4. Behavioral Tests

On the last day of the experiment (Day 35), 24 h after last MnCl_2_ dose for each group, animals’ behavioral tests, including open-field test, swimming test, catalepsy tests, such as grid test and bar test, together with Y-maze test were conducted.

#### 4.4.1. Open Field

Behavioral measures including locomotor activity, latency, and rearing are sensitive to varying degrees of DA loss in the striatum [69]. Rats were placed individually in the middle square of open-field box made of wood with the measurements (80 × 80 × 40 cm), with red walls and white smooth polished floor, divided by black lines into 16 equal squares 4 × 4 [70]. A video camera was fixed on the top of the box. Rats were allowed to freely explore the area for 3 min using a stopwatch. Behavioral changes were measured and analyzed using: latency time (the time from placing the rat in the middle of the arena until it decided to move), rearing frequency (the number of times the rat stood stretched on its hind limbs with and without forelimbs support), grooming time (the time spent scratching the rat’s face, licking forelimbs, fur, and genitals during 3 min period), and ambulation frequency (the number of squares crossed by the rat during 3 min period) [71,72].

#### 4.4.2. Swimming Test

The swimming test for assessment of motor coordination was performed as described previously [73,74]. Briefly, the apparatus used consisted of a glass tank (91.4 cm × 91.4 cm × 30.5 cm) half-filled with water and the temperature was adjusted at 26–27 °C using a thermostat. The stainless steel ramp was stabilized at the middle of one side of the glass tank while the swimming starting point was at the middle of the opposite side of the tank. Rats were taken to the test situation one hour before the test; they were placed individually at the starting point and observed until reaching the ramp for a maximum of 3 min. The behavior of rats in the swimming apparatus was evaluated by the following parameters:

##### Latency Time

The time from dropping the rat into the water till it starts swimming, measured in seconds using a stopwatch.

##### Swimming Time

The time taken to swim from starting point till reaching the ramp with forepaws. This was measured in seconds using a stopwatch.

##### Swimming Direction Score

This ranged from (0–4) as follows:

- Score (4): when the rat swims straight from the starting point to the ramp.
- Score (3): when the rat reaches the ramp through either right or left direction.
- Score (2): when the rat reaches the ramp through both right and left directions.
- Score (1): when the rat swims in all directions and in the middle but finally reaches the ramp during the 3 min.
- Score (0): when the rat swims in all directions and floats passively in the water but cannot reach the ramp within 3 min.

The measured parameters were recorded as index of muscular strength, neuromuscular coordination, as well as awareness and vigilance.

#### 4.4.3. Catalepsy Test

Catalepsy test was performed to qualify and quantify bradykinesia, akinesia, and rigidity as motor manifestations of Parkinson’s disease, as this test consists of placing the rat into an unusual posture and recording the time taken by the rat to correct this posture. This time is regarded as an index of the intensity of catalepsy [75]. Two tests of catalepsy are commonly used: the vertical grid test and the horizontal bar test.

#### 4.4.4. Grid Test

The rat was hung by its four paws on a vertical stainless steel mesh (50 cm × 40 cm, the distance between mesh weave: 0.9 cm × 1.7 cm) with a wooden frame, and the time for the rat to move its paws or show first movement was recorded.

#### 4.4.5. Bar Test

Rats were placed individually with both forepaws on a bar (9 cm above and parallel from the base) in a half-rearing position, and the time taken to remove one or both paw(s) was recorded.

#### 4.4.6. Y-Maze Test

This test is used for assessment of the ability of rats to explore new environments, where rats normally tend to discover a new maze arm rather than returning to one that was previously visited. The results of this test represent spatial working memory, which is a type of short-term memory. The used Y-maze apparatus was a wooden, black maze with three equal-sized arms in the form of a capital Y, labeled A, B, and C, respectively. Each arm (12 cm width, 40 cm length, 35 cm height) was oriented at an angle of 120° from the other two arms [76]. During the test, each rat was placed in the center of the Y maze, then the sequence of entries into the three arms was recorded over a period of 8 min. A valid arm entry was considered when the hind paws of the rat were fully entered within the arm. The ability to alternate requires the rat to memorize which arms have been entered previously. Spontaneous alternation behavior was identified as entry into all three arms on successive choices. Each experiment was scored, and the percentage of spontaneous alternation (SAP) was calculated using the following equation, according to [76,77]: SAP = [(number of alternations)/(total arm entries−2)] × 100. The floor was cleaned with 10% ethanol and then dried with a clean cloth before the entry of the next rat.

### 4.5. Tissue Sampling and Preparation

24 h following the behavioral tests, rats were anesthetized by injection of ketamine (80 mg/kg, i.p.) and sacrificed by decapitation, as previously described by Abu-Elfotuh et al. [41]. The brains were removed for postmortem biochemical and histological assessments. Brains were dissected and washed with saline. Four brains from each group were immediately fixed in 10% neutral buffered formalin for histopathological examinations. The remaining brains were divided into three portions. The first portion was homogenized separately in ice-cold PBS (pH = 7.4) to obtain a 10% homogenate (*w*/*v*). The homogenate was centrifuged at 1800× *g* for 10 min. at 4 °C, the supernatant was used for the analyses of the following biochemical parameters: brain neurotransmitter levels (dopamine (DA)), norepinephrine (NE), serotonin (5-HT), γ-aminobutyric acid (GABA and glutamate), brain derived neurotrophic factor (BDNF), inducible nitric oxide synthase (iNOS), tumor necrosis factor alpha (TNF-α), TLR4, NF-κB, NLRP3, IL-1β, COX-2, caspase-1, caspase-3, Nrf2, HO-1, malondialdehyde (MDA), and total antioxidant capacity (TAC), together with acetylcholine esterase (ACHE) and superoxide dismutase (SOD) activities. The second and third portions were kept at −80 °C, and were later used for real-time PCR (RT-PCR) analyses.

### 4.6. Colorimetric Estimation of Oxidative Stress Biomarkers; Malondialdehyde (MDA), Total Antioxidant Capacity (TAC), and Superoxide Dismutase (SOD)

Estimating the content of MDA (Cat No MD 25 28), activity of SOD (Cat No SD 25 20), and the level of TAC (Cat No TA 25 12) in brain tissue homogenate was carried out using commercially available kits obtained from Biodiagnostic, Giza, Egypt, according to the manufacturer’s instructions.

### 4.7. Fluorometric Assay of Neurochemical Markers; Norepinephrine (NE), Dopamine (DA), and Serotonin (5 HT)

As previously described by Abu-Elfotuh et al. [41] briefly, brain monoamines (NE, DA, and 5 HT) were immediately detected fluorometrically, using the commercially available fluorometric kits (Sigma-Aldrich Co., St. Louis, MO, USA), according to the manufacturer’s instructions. Monoamines were detected by specific fluorescence at specific wavelengths of excitation and emission using Hitachi (F3010 model) spectrophotofluorometer [78]. The obtained fluorescence is read at wavelengths 320 nm and 480 nm for excitation and emission, respectively, for dopamine, wavelengths 380 nm and 480 nm for excitation and emission, respectively, for NE, and wavelengths 355 nm and 470 nm for excitation and emission, respectively, for serotonin.

### 4.8. Enzyme-Linked Immunosorbent Assays (ELISA)

As previously described in [41], brain ACHE activity, as well as brain GABA, glutamate, and BDNF were estimated by commercial ELISA kits that were obtained from MyBioSource, San Diego, CA, USA. Moreover, brain TNF-α, iNOS, TLR4, NF-κB, NLRP3, IL-1β, COX-2, caspase-1, caspase-3, Nrf2, and HO-1 contents were determined by commercial ELISA kits that were obtained from Sunlong Biotech Co., Zhejiang, China. All these parameters were detected in the supernatant of brain tissue homogenate 10%, according to the manufacturer’s instructions.

### 4.9. Real-Time Quantitative Polymerase Chain Reaction (RT-qPCR)

Brain gene expression of *Bax*, *Bcl2, AIF, GFAB*, and *Gsk-3β* were estimated using qRT-PCR analysis. Total RNA was extracted using SV Total RNA Isolation System (Promega, WI, USA), reverse transcribed using the SuperScript II Reverse Transcriptase kit (Invitrogen, CA, USA), and qPCR was performed using SYBR Green PCR Master Mix (Applied Biosystems, CA, USA), according to the manufacturer’s protocol. The temperature cycling included heating at 94 °C for 40 s (step of denaturation). Then, the multiplication phase is following this step for 5 s at 95 °C and 25 s at 60 °C for 40 cycles, and melting curve analysis ramping from 65 °C to 95 °C and rinsing 1 °C each step. Samples were normalized to glyceraldehyde-3 phosphate dehydrogenase (GAPDH) expression. The relative expression of target genes (*Bax*, *Bcl2*, apoptosis inducing factor (AIF), glial fibrillary acidic protein (GFAP), and glycogen synthase kinase-3β (GSK-3β) was obtained using the following formula: 2^−∆∆CT^ [79,80]. The utilized primer sequences for the PCR amplification of *Bax*, *Bcl2*, *AIF*, *GFAP*, and *GSK-3β* are depicted in Table 5.

### 4.10. Histopathological Examinations

Brain specimens were fixed in 10% formalin for 24 h, then washed with tap water. For light microscopy, the specimens were prepared and stained according to the method described by [80]. Serial dilutions of alcohol (methyl, ethyl, and absolute ethyl) were used for dehydration. Specimens were cleared in xylene embedded in paraffin at 56 °C in hot air oven for 24 h. Paraffin beeswax tissue blocks were prepared for sectioning at 4 microns thickness by microtome. The obtained tissue sections were collected on glass slides and deparaffinized. After that, sections were stained with Hematoxylin and Eosin stain for routine histological examination through the light electric microscope at a magnification power of 40×.

### 4.11. Statistical Analysis

Data were initially tested for normality using Shapiro–Wilk’s test, with data being normally distributed if *p* > 0.05. Data were expressed as the mean + S.E.M. Multiple comparisons were performed using one-way ANOVA followed by Tukey–Kramer as a post hoc test. Differences were considered significant at *p* < 0.05. All statistical analyses were performed, and graphs were sketched, using GraphPad Prism (ISI, USA) software (version 5) computer program. The effects of treatment with nutraceuticals; sesamol, thymol, WG, CoQ10, or their combination, in normal animals are not shown to avoid complexity of the data. There were no significant differences between normal control group and nutraceuticals-treated normal animals.

## 5. Conclusions

Pretreatment with a combination of sesamol, thymol, CoQ10, and WG ameliorated MnCl_2_-induced perturbations in motor coordination, cognitive functions, histopathological findings, as well as molecular and biochemical signals of the brain, through halting oxidative damage, neuroinflammation, and neurodegeneration, via modulating TLR4/NLRP3/NF-κB, GSK-3β, Nrf2/HO-1, and apoptotic signaling pathways; summarized in (Graphical Abstract). Of note, this study depicted also that sesamol or thymol showed better protection against neuronal degeneration and some behavioral impairments than WG, or CoQ10. However, compared to monotherapy, the combined regimen had stronger neuroprotective effects in the majority of measured parameters and preserved the normal histological picture of the brain, highlighting a potential synergistic effect between these nutraceuticals in improving Parkinson’s disease. Hence, it could be regarded as a promising avenue to treat or delay the progression and/or improve the quality of life of PD patients.

## Figures and Tables

**Table 1:** Effect of sesamol, thymol, CoQ10, WG, or their combination on MnCl2-induced changes in brain monoamine levels and ACHE activity.

Groups	DA	NE	5HT	ACHE
	(ng/g Tissue)	(nmol/g Tissue)	(ng/g Tissue)	(U/L)
**Control**	72.2 ± 2.7	574.5 ± 7.9	11.2 ± 0.3	24.9 ± 1.2
**Mn**	19.1 ± 0.75 ^a^	190.3 ± 3.1 ^a^	3.6 ± 0.2 ^a^	89.6 ± 2.5 ^a^
**Mn + Sesamol**	31.8 ± 0.9 ^abc^	373 ± 12.7 ^abc^	7.3 ± 0.2 ^ab^	52.8 ± 0.8 ^abc^
**Mn + Thymol**	45.1± 0.9 ^abcd^	372.7 ± 2.5 ^abc^	6.1 ± 0.3 ^abc^	55.9 ± 2.2 ^abc^
**Mn + CoQ10**	25.6 ± 1.1 ^ace^	359 ± 5.7 ^abc^	5.7 ± 0.2 ^abcd^	59.1 ± 1.1 ^abc^
**Mn + WG**	35.2 ± 2.1 ^abcef^	359 ± 5.7 ^abc^	5.5 ± 0.2 ^abcd^	59.08 ± 1.0 ^abc^
**Mn + COMB**	62.0 ± 1.1 ^ab^	453.9 ± 14.1 ^ab^	7.9 ± 0.2 ^ab^	36.5 ± 1.3 ^ab^

Values are means of 8 rats ± S.E.M, as compared with control (a), Mn (b), COMB (c), sesamol (d), thymol (e), and CoQ10 (f) groups. One-way ANOVA with posttest Tukey’s multiple comparison assessed the statistical differences between the various groups, *p*-value < 0.05.

**Table 2:** Effect of sesamol, thymol, CoQ10, WG, or their combination on MnCl_2_-induced changes in brain BDNF, GABA, glutamate, and COX-2 levels.

Groups	BDNF	Glutamate	GABA	COX2
	(U/g Tissue)	(ng/g Tissue)	(ng/g Tissue)	(ng/g Tissue)
**Control**	156.9 ± 2.6	1.1 ± 0.06	46.8 ± 0.8	11.2 ± 44
**Mn**	59.3 ± 2.3 ^a^	8.4 ± 0.6 ^a^	11.2 ± 0.4 ^a^	46.77 ± 0.85 ^a^
**Mn + Sesamol**	117.8 ± 1.4 ^abc^	4.1 ± 0.2 ^ab^	18.2 ± 0.6 ^abc^	20.5 ± 0.48 ^abc^
**Mn + Thymol**	119.1 ± 0.38 ^abc^	4.8 ± 0.1 ^ab^	21.7 ± 0.6 ^abcd^	21.7 ± 0.56 ^abc^
**Mn + CoQ10**	119.0 ± 1.4 ^abc^	5.2 ± 0.3 ^ab^	17.4 ± 0.6 ^abce^	21.8 ± 69 ^abc^
**Mn + WG**	101.0 ± 3.2 ^abcdef^	5.2 ± 0.3 ^ab^	17.4 ± 0.6 ^abce^	22.1 ± 0.50 ^abc^
**Mn + COMB**	135.3 ± 1.8 ^ab^	3.9 ± 0.1 ^ab^	29.9 ± 0.7 ^ab^	29.9 ± 0.47 ^ab^

Values are means of 8 rats ± S.E.M, as compared with control (a), Mn (b), COMB (c), sesamol (d), thymol (e), and CoQ10 (f) groups. One-way ANOVA with posttest Tukey’s multiple comparison assessed the statistical differences between the various groups, *p*-value < 0.05.

**Table 3:** Effect of sesamol, thymol, CoQ10, WG, or their combination on MnCl_2_-induced changes in brain redox status.

Groups	TAC	SOD	MDA	iNOS
	(µmol/g Tissue)	(U/g Tissue)	(nmol/g Tissue)	(U/mg Protein)
**Control**	46.8± 0.8	7.1 ± 0.5	8.7 ± 0.6	1.6 ± 0.07
**Mn**	8.9 ± 0.3 ^a^	0.4 ± 0.01 ^a^	115 ± 0.7 ^a^	43.8 ± 0.9 ^a^
**Mn + Sesamol**	26.9 ± 0.8 ^abc^	3.5 ± 0.2 ^abc^	63.8 ± 3.6 ^abc^	14.1 ± 0.6 ^abc^
**Mn + Thymol**	21.7 ± 0.6 ^abcd^	3.3 ± 0.07 ^abc^	56.1 ± 1.5 ^ab^	14.6 ± 0.5 ^abc^
**Mn + CoQ10**	22.7 ± 1.8 ^abc^	2.8 ± 0.08 ^abc^	81.3 ± 1.4 ^abcde^	18.3 ± 0.6 ^abcde^
**Mn + WG**	18.2 ± 0.6 ^abcd^	2.9 ± 0.1 ^abc^	79.6 ± 2.3 ^abcde^	21.2 ± 0.7 ^abcdef^
**Mn + COMB**	33.2 ± 1.8 ^ab^	4.9 ± 0.06 ^ab^	48.3 ± 2.7 ^ab^	7.2 ± 0.7 ^ab^

Values are means of 8 rats ± S.E.M, as compared with control (a), Mn (b), COMB (c), sesamol (d), thymol (e), and CoQ10 (f) groups. One-way ANOVA with posttest Tukey’s multiple comparison assessed the statistical differences between the various groups, *p*-value < 0.05.

**Table 4:** Experimental design (Groups and doses).

Group	Dose Regimen	Reference
I (Control)	Received saline with 1% tween 80 for 5 weeks and served as the vehicle-treated normal control.	
II (Mn)	Injected with MnCl_2_ (10 mg/kg/day, i.p.) for 5 weeks.	[32]
III (Mn + Sesamol)	Treated with sesamol (15 mg/kg/day, p.o.) 1 h before Mn (10 mg/kg/day, i.p.) for 5 weeks.	[34]
IV (Mn + Thymol)	Treated with thymol (30 mg/kg/day, p.o.) 1 h before Mn (10 mg/kg/day, i.p.) for 5 weeks.	[67]
V (Mn + CoQ10)	Treated with CoQ10 (200 mg/kg/day, p.o.) 1 h before Mn (10 mg/kg/day, i.p.) for 5 weeks.	[35]
VI (Mn + WG)	Treated with WG (100 mg/kg/day, p.o.) 1 h before Mn (10 mg/kg/day, i.p.) for 5 weeks.	[68]
VII (Mn + COMB)	Treated with sesamol (15 mg/kg), thymol (30 mg/kg), CoQ10 (200 mg/kg), and WG (100 mg/kg) as the same aforementioned doses, 1 h before Mn (10 mg/kg/day, i.p.) for 5 weeks.	

Mn: Manganese Chloride, WG: Wheatgrass, COMB: sesamol (15 mg/kg), thymol (30 mg/kg), CoQ10 (200 mg/kg), and WG (100 mg/kg).

**Table 5:** Primer sequences for Real-Time RT-PCR Analysis.

Target	Primer Sequence	Accession Numbers	Reference
*Bcl-2*	F: 5′-GGATGACTTCTCTCGTCGCTAC-3′	NM_016993	[81]
	R: 5′-TGACATCTCCCTGTTGACGCT-3′		
*Bax*	F: 5′-CACGTCTGCGGGGAGTCA-3′	NM_017059	[60]
	R: 5′-TAGGAAAGGAGGCCATCCCA-3′		
*AIF*	F: AGTCCTTATTGTGGGCTTATCAAC	NM_031356	[82]
	R:TTGGTCTTCTTTAATAGTCTTGTAGGC		
*GFAP*	F: ACAGACTTTCTCCAACCTCCAG	NM_017009	[82]
	R: CCTTCTGACACGGATTTGGT		
*GSK-3β*	F: 5′-AGCCTATATCCATTCCTTGG-3′	NM_032080	[41]
	R: 5′-CCTCGGACCAGCTGCTTT-3′		
GAPDH	F: 5’-GGGCAGCCCAGAACATCA-3’	NM_017008	[81]
	R: 5’-TGACCTTG CCCACAGCCT-3’		

F: Forward primer, R: Reverse primer.

## Data Availability

Data is contained within the article and other data will be available upon request.

## Abbreviations

ACHE; acetylcholine esterase, AIF; apoptosis inducing factor, Bax; B-cell lymphoma protein 2 (Bcl2)- associated X protein, Bcl2; B-cell lymphoma-2 protein, BDNF; brain-derived neurotrophic factor, CoQ10; coenzyme-Q10, GABA; γ-aminobutyric acid, GFAP; glial fibrillary acidic protein, GSK-3β; glycogen synthase kinase-3β, DA; dopamine, iNOS; inducible nitric oxide synthase, i.p.; intraperitoneal, Keap-1; Kelch-like ECH-associated protein 1, MDA; malndialdehyde, Mn; manganese, NE; norepinephrine, NF-κB; nuclear factor kapa B, Nrf2/HO-1; nuclear factor erythroid 2-related factor 2/hemoxygenase-1, PD; Parkinson’s disease, SAP; spontaneous alternation percentage, SOD; superoxide dismutase, TAC; total antioxidant capacity, TLR-4; Toll-like receptor-4, TNF-α; tumor necrosis factor alpha, WG; wheatgrass, 5-HT; serotonin.
